# A Pilot Study of the Relationship between Diet and Mental Health in Community Dwelling Japanese Women

**DOI:** 10.3390/medicina55090513

**Published:** 2019-08-21

**Authors:** Naoko Takashima, Akihiko Katayama, Kazumi Dokai Mochimasu, Shuhei Hishii, Hiromi Suzuki, Nobuyuki Miyatake

**Affiliations:** 1 Department of Hygiene, Faculty of Medicine, Kagawa University, Kita-gun, Kagawa 761-0793, Japan; 2 The Faculty of Sociology, Shikoku Gakuin University, Zentsuji, Kagawa 765-8505, Japan; 3 Department of Food Science, Mimasaka University, Tsuyama, Okayama 708-8511, Japan

**Keywords:** nutrients, n-6/n-3 fatty acid ratio, brief-type self-administered diet history questionnaire (BDHQ), mental health, general health questionnaire-12 (GHQ), women

## Abstract

*Background and Objectives*: Diet is closely linked to mental health. The aim of this study was to explore the link between diet and mental health in community dwelling Japanese women. *Materials and Methods*: A total of 89 community dwelling Japanese women, aged 66.8 ± 7.6 years, were enrolled in this cross-sectional study. Diet surveys were evaluated using the brief-type self-administered diet history questionnaire (BDHQ) and mental health was evaluated using the general health questionnaire-12 (GHQ) and clinical parameters. *Results*: The mean energy intake was 1806 ± 570 kcal and the GHQ score was 1.0 ± 1.4. Among nutrients, the n-6/n-3 fatty acid ratio was significantly correlated with the GHQ score (r = 0.269, *p* = 0.011), and some micronutrients and vitamins were weakly or negatively correlated with the GHQ score. Among the food groups, fish and shellfish were negatively correlated with the GHQ scores. Multiple regression analysis demonstrated that the n-6/n-3 fatty acid ratio was the determinant factor for the GHQ score, even after adjusting for confounding factors. *Conclusions*: These results suggest that a proper diet to reduce the n-6/n-3 fatty acid ratio may improve mental health in community dwelling Japanese women.

## 1. Introduction

Proper management of mental health has become a public health challenge in Japan [1,2]. Therefore, several strategies to improve mental health, such as minor psychiatric disorders, have been employed in workplaces, schools and communities in Japan [3].

It is well known that mental health is affected by lifestyle, such as physical activity [4,5], cigarette smoking [6], alcohol drinking [7,8], sleep disturbances [9,10] and diet [11,12,13,14,15,16,17,18,19,20,21,22], as well as by medications [23,24]. Although there are some reports of the relationship between diet and mental health [11,12,13,14,15,16,17,18,19,20,21,22], evaluation of diet surveys is not accurate or reliable, as expected. In addition, we previously investigated the link between diet and mental health in female university students enrolled in a training course for registered dietitians, who were thought to be more health conscious than average students [25], and found that the consumption of confections is associated with mental health [25]. However, in other ages, accurately and reliably evaluated, the link between diet and mental health remains to be investigated.

Therefore, in this pilot cross-sectional study, we explored the relationship between diet (nutrients and food groups) and mental health in community dwelling Japanese women.

## 2. Materials and Methods

This is a secondary analysis study. Participants voluntarily took part in the previously reported randomized controlled trial (RCT) of an exercise program who lived around Seto Inland Sea, Western Japan [26]. The primary outcome was the general health questionnaire-12 (GHQ-12) between the two groups with the difference (0.7) and standard deviation (SD) (1.2) (α = 0.05, β = 0.20, dropout rate = 10%). The sample size of the first study was 120. A total of 89 community dwelling Japanese women (74.1%) among baseline data of 120 men and women, with a mean age of 66.8 ± 7.6 years, who met the following criteria were enrolled in this secondary study (Table 1): (1) they underwent the survey of diet and mental health, and (2) they provided written informed consent.

Clinical parameters, such as age, height (cm), body weight (kg), body fat percentage (%), diet and mental health, were examined. Body mass index (BMI) was calculated as follows: Body weight (kg)/[height (m)]^2^. Body fat percentage (%) was measured using a professional multifrequency body composition meter (MC-180, Tanita Co Ltd., Tokyo, Japan) [27].

Diet was evaluated using the brief-type self-administered diet history questionnaire (BDHQ), which can accurately and reliably estimate the intake of nutrients and food groups, as previously described [28,29,30,31].

Mental health was evaluated using GHQ-12, which is well prepared and tested with good validity and reliability as previously described [32,33,34,35]. This questionnaire is a screening device for identifying minor psychiatric disorders in the general population, and it is a good screener of short-term psychological wellbeing rather than just a unitary screening measure. GHQ-12 scores were determined according to the original method (0-0-1-1) [35].

Data were expressed as the mean ± SD. Simple correlation analysis was used to evaluate the relationship between nutrients and the GHQ score, and between food groups and the GHQ score, where *p* < 0.05 was significant. Multiple linear regression analysis was used to evaluate which factor among the nutrients was important for the GHQ score in community dwelling Japanese women.

Ethical approval was obtained from the ethics committee of Shikoku Gakuin University (Approved number: 2015001, Date: 26 May 2015).

## 3. Results

The clinical profiles of enrolled women are summarized in Table 1. The mean age, height (cm), body weight (kg), BMI (kg/m^2^) and body fat percentage (%) were 66.8 ± 7.6 years, 153.6 ± 5.4 cm, 52.8 ± 6.8 kg, 22.4 ± 2.6 kg/m^2^ and 28.8 ± 6.3%, respectively. The mean GHQ score was 1.0 ± 1.4.

The daily intake of nutrients and food groups is summarized in Table 2 and Table 3. Among the nutrients, the mean energy intake was 1806 ± 570 kcal. Protein, fat and carbohydrate intakes were 81.0 ± 34.0 g, 59.3 ± 22.9 g and 230.3 ± 76.2 g, respectively. The mean n-6/n-3 fatty acid ratio was 3.6 ± 0.8 (Table 2). Among the food groups, the mean intake of fish and shellfish was 109.4 ± 74.8 g (Table 3).

**Figure 1:**
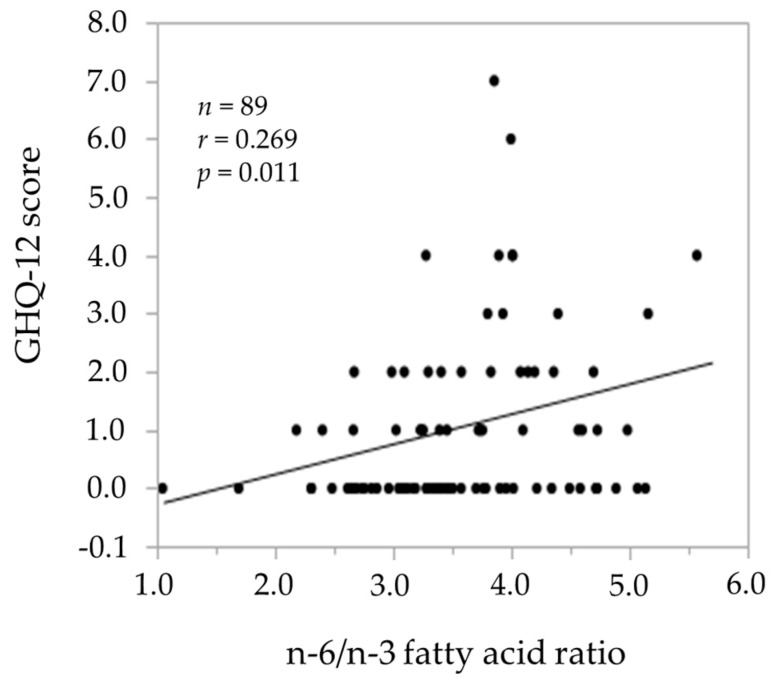
The relationship between the n-6/n-3 fatty acid ratio and the GHQ-12 score.

We next evaluated the relationship between nutrients and the GHQ score using simple correlation analysis (Table 4). Among the nutrients, the n-6/n-3 fatty acid ratio was significantly and positively correlated with the GHQ scores (r = 0.269, *p* = 0.011) (Figure 1). Magnesium, calcium, vitamin D, niacin and vitamin B_12_ were weakly or negatively (r = −0.209~−0.233) correlated with the GHQ score. Among the food groups (Table 5), fish and shellfish were negatively correlated with the GHQ score (r = −0.233, *p* = 0.027).

Lastly, we examined which nutrients were important for the GHQ score by multiple linear regression analysis (Table 6). We used the GHQ scores as the dependent valuable, and age (years), BMI (kg/m^2^), n-6/n-3 fatty acid ratio and sucrose (g). The n-6/n-3 fatty acid ratio was statistically significant by simple correlation analysis in our study, and other factors were considered to be clinically important based on our previous study [25]. As a result, n-6/n-3 fatty acid ratio (β: 0.230, *p* = 0.049) was found to be a determinant factor for the GHQ scores, even after adjusting for age (years), BMI (kg/m^2^) and sucrose (g) in community dwelling Japanese women.

## 4. Discussion

There are many reports of the relationship between diet and mental health [11,12,13,14,15,16,17,18,19,20,21,22]. In a cross-sectional study, Miki et al. investigated the relationship between nutrients and mental health using the BDHQ and Center for Epidemiologic Studies Depression Scale (CES-D) in 1792 men and 214 women employees, and found that magnesium, calcium, iron and zinc were associated with depression [11]. In a cohort study, vitamin D intake was also linked to depression symptoms [12] as evaluated by the Burnam scale [13]. Vitamin B_6_, B_12_ and Folate, as evaluated by the Food Frequency Questionnaire (FFQ), were also found to be associated with depressive symptoms in community dwelling older adults [14]. In the Japan Public Health Center-based prospective Study (JPHC Study), the relationship between fish intake and depression was evaluated by the FFQ [15].

Regarding the n-6/n-3 fatty acid ratio, Beydoun et al. previously investigated the link between the n-3/n-6 fatty acid ratio, as evaluated by the 24 h dietary recall method and depressive symptoms in women in a cohort study in the USA, and found that the n-3/n-6 fatty acid ratio was closely associated with depressive symptoms [16]. Da Rocha et al. also reported the relationship between the n-6/n-3 fatty acid ratio and depression in pregnancy using the FFQ [17]. Miyake et al. found that fish intake, but not n-6/n3 fatty acid ratio, was associated with depressive symptoms during pregnancy [18]. On the other hand, Murakami et al. reported in their review that no association was found between dietary variables and depressive symptoms, because of the unreliable or rough assessment of diet or depressive symptoms [19].

In this pilot study, the intake of fish and shellfish was 109.4 ± 74.8 g. We noted the consumption of fish and shellfish was higher than that of the National Nutrition Survey (2016: 60.3 ± 57.4 g) [36], and it showed a significant relationship between nutrients and food groups, as in previous studies, especially the n-6/n-3 fatty acid ratio, and the GHQ scores in community dwelling Japanese women. We used the BDHQ, which is an accurate and reliable method for evaluating nutrients and food groups [28,29,30]. Based on the multiple regression analysis, the n-6/n-3 fatty acid ratio is a determinant factor for the GHQ score, even after adjusting for confounding factors. Eating n-3 fatty acids improved the symptoms of attention deficit hyperactivity disorder (ADHD) patients [20], and also improved anxiety symptoms in medical students [21] and depressed adult outpatients [22] by RCT. In Japan, the mean consumption of n-6 and n-3 fatty acids are 9.6 g/day and 2.1 g/day [36], respectively and the recommended n-6/n-3 fatty acid ratio is 4 by the Ministry of Health, Labour and Welfare, Japan [37]. Taken together, we recommend reducing the n-6/n-3 fatty acid intake by eating fish and shellfish to improve the mental health of community dwelling Japanese women in clinical practice.

This study has several potential limitations. First, this was a secondary cross-sectional study with small samples. Second, the voluntarily enrolled women were considered to be more health conscious than the average subjects. Third, we were unable to evaluate the relationship between diet and mental health in men because of a small sample in the first study. Fourth, we could not obtain details of sociodemographic characteristics such as educational level, socioeconomic status, marital status, the number of people living in the household with the study subjects, and medical history to adjust the relationship between diet and mental health. However, diet is thought to be a modifiable risk factor contributing to an individual’s mental health and, in combination with other interventions of an individual’s lifestyle factors, may be the main current approach for preventing and improving mental health. Fifth, the mechanism of the correlation between n-6/n-3 fatty acid ratio and the GHQ score was not evaluated. However, a proper diet, which includes an improved n-6/n-3 fatty acid ratio by eating fish and shellfish, may improve the mental health of community dwelling Japanese women. Further prospective and intervention studies are required in the future. 

## 5. Conclusions

In this pilot cross-sectional study, we evaluated the link between diet and mental health in community dwelling Japanese women, and we found that some nutrients and food groups, especially the n-6/n-3 fatty acid ratio, were closely associated with the GHQ score.

## Figures and Tables

**Table 1:** Characteristics of the enrolled women.

	Mean	±	SD
Age (years)	66.8	±	7.6
Height (cm)	153.6	±	5.4
Body weight (kg)	52.8	±	6.8
BMI (kg/m^2^)	22.4	±	2.6
Body fat percentage (%)	28.8	±	6.3
GHQ score	1.0	±	1.4

BMI: body mass index. GHQ: general health questionnaire-12.

**Table 2:** Daily intake of energy and nutrient measured using the BDHQ.

	Mean	±	SD
Energy (kcal)	1806	±	570
Protein (g)	81.0	±	34.0
Fat (g)	59.3	±	22.9
Animal fat (g)	30.5	±	14.5
Vegetable fat and oil (g)	28.9	±	11.0
Carbohydrates (g)	230.3	±	76.2
Sodium (mg)	4370	±	1483
Magnesium (mg)	300	±	111
Calcium (mg)	707	±	323
Iron (mg)	9.4	±	3.5
Vitamin D (μg)	21.0	±	15.8
Vitamin A (Retinaol activity equivalents) (μg)	958	±	567
Vitamin B_1_ (mg)	0.91	±	0.36
Vitamin B_2_ (mg)	1.59	±	0.58
Vitamin C (mg)	150	±	71
Niacin (mg)	20.7	±	9.5
Vitamin B_12_ (mg)	13.3	±	8.3
Total dietary fiber (g)	14.6	±	5.6
Salt equivalents (g)	11.0	±	3.7
Sucrose (g)	11.6	±	9.3
n-3 fatty acid (g)	3.2	±	1.4
n-6 fatty acid (g)	10.8	±	3.6
n-6/n-3 fatty acid ratio	3.6	±	0.8

BDHQ: brief-type self-administered diet history.

**Table 3:** Daily intake of food groups measured using the BDHQ.

	Mean	±	SD
Cereals (g)	337.3	±	159.0
Potatoes (g)	50.8	±	40.9
Sugars and sweeteners (g)	4.7	±	2.9
Pulses (g)	82.8	±	43.7
Green and yellow vegetables (g)	146.3	±	81.7
Other vegetables (g)	221.6	±	120.3
Fruits (g)	153.1	±	96.0
Fish and shellfish (g)	109.4	±	74.8
Meats (g)	76.7	±	53.7
Eggs (g)	45.7	±	21.6
Milk (g)	175.1	±	105.2
Fats and oils (g)	10.3	±	5.2
Confections (g)	45.0	±	40.6
Beverages (g)	689.5	±	328.1
Seasonings and spices (g)	206.9	±	149.4

BDHQ: brief-type self-administered diet history.

**Table 4:** Simple correlation analysis between nutrients and GHQ score.

	r	*p*
Energy (kcal)	−0.086	0.420
Protein (g)	−0.171	0.108
Fat (g)	−0.065	0.543
Animal fat (g)	−0.182	0.087
Vegetable fat and oil (g)	−0.075	0.484
Carbohydrates (g)	−0.033	0.754
Magnesium (mg)	−0.213	**0.044**
Calcium (mg)	−0.233	**0.027**
Iron (mg)	−0.162	0.129
Vitamin D (μg)	−0.229	**0.031**
Vitamin A (Retinol activity equivalent) (μg)	−0.127	0.233
Vitamin B_1_ (mg)	−0.137	0.199
Vitamin B_2_ (mg)	−0.172	0.105
Vitamin C (mg)	−0.167	0.115
Niacin (mg)	−0.209	**0.048**
Vitamin B_12_ (mg)	−0.219	**0.039**
Total dietary fiber (g)	−0.139	0.192
Salt equivalents (g)	−0.163	0.125
Sucrose (g)	−0.032	0.763
n-3 fatty acid (g)	−0.185	0.082
n-6 fatty acid (g)	−0.033	0.755
n-6/n-3 fatty acid ratio	0.269	**0.011**

Bold values are significant. (*p* < 0.05). GHQ: general health questionnaire-12.

**Table 5:** Simple correlation analysis between food groups and GHQ score.

	r	*p*
Cereals (g)	0.037	0.727
Potatoes (g)	−0.098	0.357
Sugars and sweeteners (g)	−0.115	0.279
Pulses (g)	−0.091	0.395
Green and yellow vegetables (g)	−0.165	0.121
Other vegetables (g)	−0.082	0.440
Fruits (g)	−0.162	0.129
Fish and shellfish (g)	−0.233	**0.027**
Meats (g)	−0.029	0.783
Eggs (g)	0.012	0.904
Milk (g)	−0.153	0.149
Fats and oils (g)	0.019	0.855
Confections (g)	0.011	0.911
Beverages (g)	−0.182	0.086
Seasonings and spices (g)	0.016	0.881

Bold values are significant. (*p* < 0.05). GHQ: general health questionnaire-12.

**Table 6:** Multiple linear regression analysis between clinical parameters and GHQ score.

	β	*p*
Dependent Variable: GHQ score		
Independent variables		
Age (years)	−0.114	0.382
BMI (kg/m^2^)	0.051	0.624
n-6/n-3 fatty acid ratio	0.230	**0.049**
Sucrose (g)	−0.060	0.565

R^2^ = 0.092, *p* = 0.082. Bold values are significant. (*p* < 0.05). BMI: body mass index. GHQ: general health questionnaire-12.
